# A Fragile Balance: Perturbation of GABA Mediated Circuit in Prefrontal Cortex Generates High Intraindividual Performance Variability

**DOI:** 10.1371/journal.pone.0005208

**Published:** 2009-04-21

**Authors:** Pierre Pouget, Nicolas Wattiez, Sophie Rivaud-Péchoux, Bertrand Gaymard

**Affiliations:** 1 Inserm U975, Movement disorders and basal ganglia, Hôpital de la Salpêtrière, Paris, France; 2 Université Pierre & Marie Curie-Paris 6, CNRS UMR 7225, Centre de Recherche – Institut du Cerveau et de la Moelle – CR-ICM, UMR S975, Paris, France; 3 AP-HP, Hôpital de la Salpêtrière, Service d'Explorations Fonctionnelles Neurologiques, Paris, France; L'université Pierre et Marie Curie, France

## Abstract

High intraindividual performance variability is one of the most robust findings to emerge in cognitive-experimental research of attention deficit hyperactivity disorder (ADHD). Evidences from studies incorporating structural or functional human brain mapping methods indicate that this increased intraindividual variability is not simply a sequel of general brain dysfunction, but is likely related to the functioning of neural circuits that engage the prefrontal cortex, particularly the dorsolateral areas (dlPFC). In order to examine further the anatomical and pharmacological substrate responsible for this high intraindividual variability disorder, we injected GABA_A_ antagonist (bicuculline) or GABA_A_ agonist (muscimol) in the dlPFC of monkeys performing a reflexive oculomotor task. Here we show that, whereas GABA_A_ agonist injection induced no or minimal impairments, injection of GABA_A_ antagonist dramatically increased the intraindividual variability of saccade response time and of saccade spatial accuracy (amplitude and direction). Overall, this study demonstrates that the balance between excitatory/inhibitory activities in the dlPFC is fragile but crucial, since local micro-injection of GABA_A_ antagonist can induce marked behavioural effects. It also reveals that higher cognitive areas such as the dlPFC are markedly capable to influence the productions and metrics of reflexive movements. Altogether, this study provides promising perspectives for the development of new therapeutic strategies for the treatment of diseases in which high intravariability disorders are a prominent feature.

## Introduction

Significant and reliable differences in the speed and the variability of responses have been documented between ADHD and typically developing children (TDC) across a wide variety of neuropsychological tasks [1], [2]. Increased variability, that is hypothesized to reflect underlying unstable attentional resources, is seen in tasks requiring continual responses to rapid stimuli as well as basic reaction time (RT) tasks [3]. Children with ADHD display are often found to respond more slowly and less accurately than the typically developing peers. However and importantly, response variability correlates more strongly and reliably with ratings of ADHD symptoms than commission errors or other outcome measures [4]. Evidences from studies incorporating structural or functional human brain mapping methods indicate that this intraindividual variability may not simply be a sequel of general brain dysfunction, but may likely be related to the functioning of neural circuits that, among other brain areas, engage the prefrontal cortex, particularly the dorsolateral areas (dlPFC) [5], [6]–[14]. In order to examine further the anatomical and pharmacological substrate of this high intraindividual variability disorder, we injected a GABA_A_ antagonist (bicuculline) or a GABA_A_ agonist (muscimol) in the dlPFC of monkeys performing a reflexive oculomotor task (see supplementary information). Here, we show that specific and focal perturbation of GABA-mediated circuit in the dlPFC is capable to generate high intraindividual performance variability.

## Results

**Figure 1:**
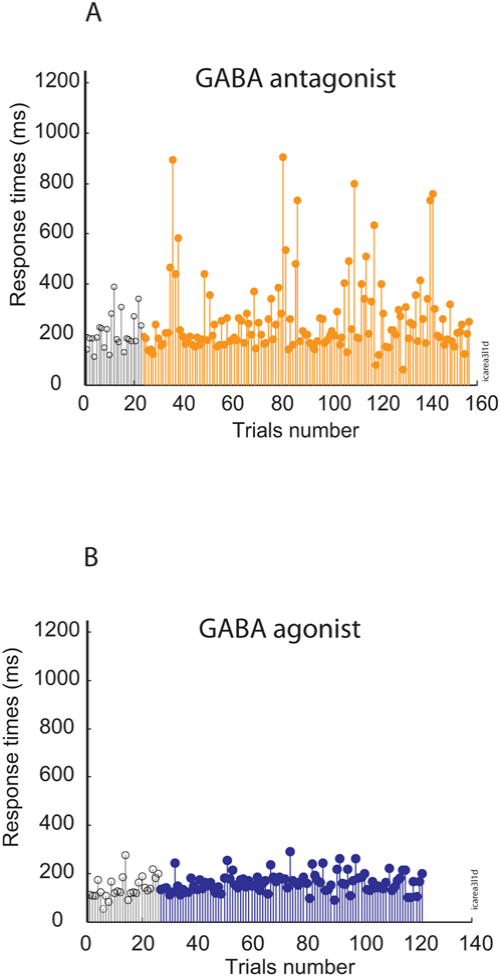
Response time sequences. (A) Response time sequences in simple pro-saccade task following GABA_A_ antagonist (bicuculline) injection. These sequences were taken from 4 consecutive blocks of trials for monkey A. (B) Same convention as A when GABA_A_ agonist was injected in the same site. Note the marked increased scatter of both parameters after bicuculline injection, with interleaved normal and markedly impaired values. Each dot corresponds to a saccade. Empty dots: pre-injection trials. Filled dots: post-injection trials.

**Figure 2:**
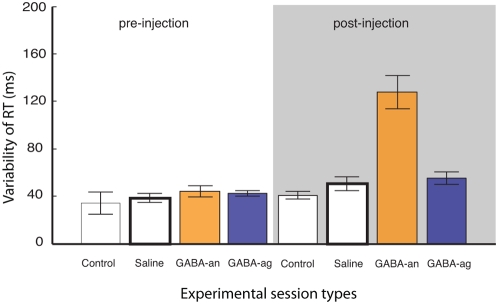
Comparison of GABA_A_ antagonist (bicuculline) and GABA_A_ agonist (muscimol) injections on response times variability (ms). Note the marked effect of GABA_A_ agonist injections and the null effect of GABA_A_ agonist on the variability of simple response times.

As shown in Figure 1, in a representative injected site within the dlPFC, the variability of simple RTs dramatically increased after GABA_A_ antagonist injection (Figure 1A), but not after GABA_A_ agonist injection (Figure 1B). This result was confirmed across all injected sites (100%; 15/15 and 11/11sites; respectively 9 and 6 for monkey A and 6 and 5 for monkey B). Statistically and compared to pre-injection periods, GABA_A_ antagonist but not GABA_A_ agonist injections significantly increased RT variability (Figure 2A and 2B) (U(15,11) = 152, *p*<0.001; and U(15,11) = 11, *p*>0.05). Similar effects were observed when complete control experiments or saline injections were compared to GABA_A_ injections (see Text S1). This effect was bilateral in both monkeys (see Figure S2 and Text S1). Injection of GABA_A_ antagonist did also affect important metric properties of the movements, since variability of both saccade gain and direction were markedly affected (see Text S1 and Figure S3, S4). Specificity of dlPFC perturbation was further tested in a third animal with a series of injections in the supplementary eye fields and Pre-SMA (Figure S6). For both SEF and Pre-SMA, the variability in response times did not significantly vary following GABA_A_ agonist or antagonist (*p*≫0.5).

**Figure 3:**
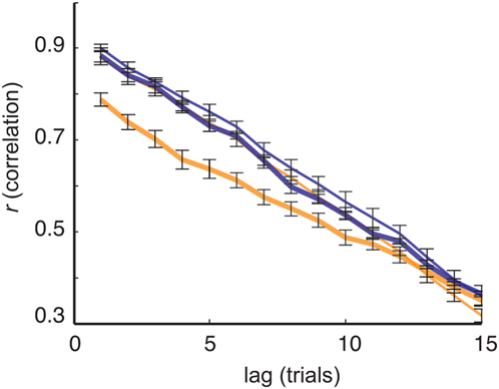
Serial correlations between response times (RTs). Average of correlations for 16 sites of monkeys A and B. The different correlations estimated from sequences preceding (thin line) or following injections (thick line) of GABA_A_ agonist (blue line) and antagonist (orange line) are plotted separately. The correlations between RTs and past RTs are presented at lags up to 15 trials.

Two methods were used to further examine the sequences of collected RTs. First, distinct serial correlations of RTs were calculated for each experimental block of trials (preceding or following the micro-injection of GABA_A_ antagonist, GABA_A_ agonist or saline). The correlations were then averaged across sessions and monkeys. Figure 3 shows the average serial correlations obtained for GABA_A_ antagonist, GABA_A_ agonist and saline injections at lags of up to 15 trials. Across monkeys and injection sites, the serial correlations obtained after GABA_A_ antagonist injections is lower than GABA_A_ agonist injections at lags up to 6 trials (U(15,11) = 336, *p* = .03 ). These results are consistent with the noticeable noise observed following GABA_A_ antagonist injection as shown in Figure 1.

**Figure 4:**
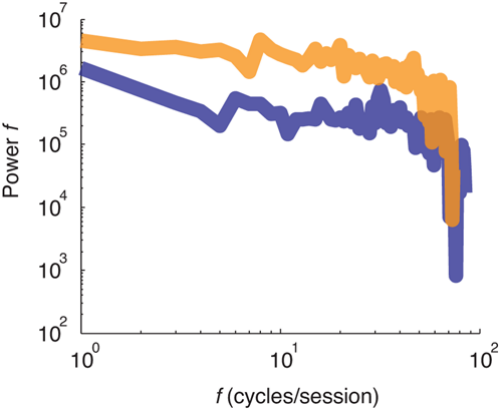
Log–log plot of average Lomb–Scargill power spectrum estimates, for response times sequences following GABA_A_ agonist (blue line) and GABA_A_ agonist (orange line) injections. Frequency f is cycles-per-block.

Then, a series of Lomb-Scargle periodograms were calculated for the different subsequences of trials following the injections. The resultant power spectra were averaged across monkeys and sessions. Similar to what has been reported previously in the literature, the power of RTs oscillations following injection of GABA_A_ agonist is centered in the lowest part of frequencies spectrum (Figure 4) c. In contrast, the power of RTs oscillations following the GABA_A_ antagonist injections are more similar to white noise power spectrum traduced by an overall increase the power spectrum distribution (Figure 4). Again, these results are consistent with the noticeable noise and the smaller lag observed in serial correlations for GABA_A_ antagonist sequences as shown in Figures 1 and 3 (see also Figure S5). Functionally, the presence of white noise within the sequences of RTs following GABA_A_ antagonist injections is also consistent with the view that these sequences were generated by a more unstable and more chaotic process in which short or long term memory are less or not expressed.

## Discussion

It is well established that simply instructing human or non-human primate subjects to focus attention is not sufficient to prevent them from distractibility [1], [2]. In the primate brain, the dlPFC is a frontal cortical region known to be engaged in these attentional, memory and high level executive functions [15], [16]. Our results show here that focal pharmacological perturbations of the dlPFC are also capable to impair a basic motor task with a low cognitive load [15]–[17]. These results are important for at least two reasons. First, they indicate that the classical boundaries delimitating the highest cognitive processes and the levels of motor encoding stages is less tight than previously thought. Second, these results open a novel route to explore long-term pharmacological treatments for the numerous patients suffering from attentional disorders.

In human, recent studies comparing TDC with ADHD children has shown increased of intraindividual variability at all parts of the spectrum of RTs, even in tasks with the lowest cognitive loads [1], [2], [10]. These measures of variability appeared to be so robust that intraindividual variability in RTs is now viewed as a valid endophenotype with the potential ability to index genetic vulnerability to ADHD reflecting attentional lapses on some but not all responses [2]. Before these recent findings, poorer performances in neuropsychological tasks of ADHD children were typically interpreted as evidence for high level executive function deficits. This interpretation was partially supported by the fact that ADHD children produce on average slower and less accurate responses than TDC. However, it has subsequently been shown that intraindividual variability of these RT sequences often correlates better with the behavioural symptoms of ADHD than the mean values. Consistent with the possibility that the dlPFC is implicated in the pathology of ADHD, we show here that minimal local micro-injections of GABA_A_ antagonist but not GABA_A_ agonist in the dlPFC exert a profound effect on intraindividual variability during sequences of a simple response task. This variability was observed in the RTs as well as the direction and the amplitude of the movement.

In our study, only Bicuculline (GABA_A_ antagonist) affected the monkey's behavior. This result may be considered as circumstantial to our simple response task but is not trivial. In V1, micro-injection of GABA_A_ antagonist (bicuculline) was found to exert a much weaker effect on neuronal responses compared to GABA_A_ agonist (muscimol) [18]. However, and importantly, it was noticed that both administrations of GABA_A_ antogonist and GABA_A_ agonist in visual cortex resulted in improved visual function [19], [20]. In other subcortical and cortical regions implicated in visual target selection and saccade programming, it has been shown that the application of GABA_A_ (bicuculline) greatly facilitates saccade production, whereas injection of GABA_A_ agonist (muscimol) inhibits saccade generation [18]–[21]. The increased intrasubject variability observed in the present study appears therefore to be specifically related to dlPFC dysfunction. Altogether these results let us speculate that, rather than the absolute activity of the GABA-mediated circuits in dlPFC, what might be more crucial to the behavioural expression depends on the fragile balance between activation or inhibition of the GABA-mediated circuits. Depending on the task, on the environmental constraints and/or the level of activity of other GABAergic circuits, a modest variation can lead to dramatic behavioural changes. In our experiment, using a simple saccadic reflexive task, a GABA_A_ antagonist action appeared to be the solely route to affect the balance and the subsequent behaviour.

The reaction time variability is one of the strongest findings that recently emerge in cognitive-experimental research on ADHD. However, it is important to notice that the symptoms of ADHD are heterogeneous and often accompanied by multiple comorbid psychiatric disorders. If our finding among others offer a new route for potential pharmacotherapies capable to influence the activity of GABA_A_ receptors, further researches would be needed in order to understand better the principles and rules that govern the variations of the most simple of our behavioural responses.

## Materials and Methods

Two adult male green monkeys (Caercopitheca aethiops) were used. The maintenance of the monkeys, all surgical procedures and the experimental protocols were carried out in strict accordance with the National Institutes of Health (NIH) guidelines (1996) and the recommendations of the EEC (86/609) and the French National Committee (87/848). Before training, each monkey underwent a surgical procedure for the implantation of a scleral search coil on each eye and an acrylic head holder. At the end of the training period, a recording chamber was implanted above the principal sulcus (PS), on each side. Heart rate and body temperature were monitored during surgery. Antibiotics and analgesic were given during the 10 following days.

### Behavioural tasks

Monkeys were trained to look at a central green fixation stimulus for 700–1,200 msec. After a 200 ms blank period (gap), a 2 deg×2 deg green target appeared 16 degrees from the fixation target at one of six possible radial locations (0°, 45°, 135°, 180°, 225°, or 315°), during 1,000 ms. After the saccade, the monkey received a reward if the saccade fell within a 5°×5° window centered on the target. Failure to enter a 3°×3° window around the fixation stimulus or to trigger a saccade within 2000 ms after target onset cancelled the trial. Each task consisted in blocks of 24 trials each in which each radial location was presented semi-randomly. Eye movements were recorded with the search coil technique, as described previously [10].

### Localization of the principal sulcus

The Figure S1 shows the injected sites. The PS was localized by electrophysiological recordings performed with tungsten microelectrodes (FHC, 8–9 MΩ at 1 kHz). Electrical stimulation (80 ms of biphasic pulses at 350 Hz, current up to 150 µA) was used to delineate the frontal eye field and confirmed that no saccade could be elicited from the posterior sites of this area.

Localization of SEF and pre-SMA. In a third control case, injections were made in the SEF and in the pre-SMA. The SEF and pre-SMA were classically localized by electrophysiological recordings performed with tungsten microelectrodes (FHC, 8–9 MΩ measured at 1 kHz). Electrical stimulation (80 ms of biphasic pulses at 350 Hz, current up to 150 µA) was used to delineate SEF and confirmed by elicitation of saccade with current as low as 50 µA. The pre-SMA was delineated centro-caudal from the posterior sites of SEF area and rostral to the orofacial region of the SMA.

### Micro-injection procedure

Bicuculline, a GABA_A_ antagonist, or muscimol (Sigma), a GABA_A_ agonist, were injected through a 30-gauge stainless steel cannula lowered into the brain with a transdural guide tube. Once the tip of the cannula was at the desired level, 2.5 µl of bicuculline (5 µg/µl) or 4 µl of muscimol (5 µg/µl) were injected with a 10 µl Hamilton syringe during 16 minutes. In monkey A, 10 sites were injected with bicuculline, four of them being injected with muscimol. In monkey B, bicuculline was injected at 6 sites, all these sites being also injected with muscimol.

### Data analysis

Saccades were identified offline and controlled visually. In each experiment, we analyzed primary saccade latency, gain and direction. Saccade gain was defined as the ratio of primary saccade amplitude and target eccentricity. Saccade direction was determined in degrees (positive values: from 0° to 180° counterclockwise, negative values: from 0° to 180° clockwise). For each saccade, we determined its directional accuracy by calculating a directional error in degrees. Thus, a +5° directional error corresponds to a saccade with a slightly counterclockwise deviated direction. The directional error was obtained by subtracting saccade direction from target direction, i.e. the ideal vector that would have been required to acquire the target.

### Statistical analyses

Differences between groups were assessed using the rank sum test, also referred as Mann-Whitney test (Matlab2008b, Mathworks). This test was used so that the normality of the populations was not assumed. Level of significance was set to *p* = 0.05. Correlations were calculated using Spearman rank order test.

## Supporting Information

Text S1 (0.02 MB DOC)

Figure S1 Injection sites. (A) Injection sites in the region of dlPFC for monkey A. Orange dots represent sites at which GABA_A_ antagonist (bicuculline) was injected. Blue circled dot represent sites at which GABA_A_ agonist (muscimol) was injected. PS: principal sulcus. AS: arcuate sulcus. (B) Same conventions for monkey B.
(0.65 MB DOC)

Figure S2 Variability of response time. (A) Variability of response times as function of experimental session types for monkey A When the eye movement is produced ipsilaterally (full line box) or contralaterally (dotted line box) to the injection site. Blue circled dots represent sites at which both bicuculline and muscimol were injected. (B) Same conventions for monkey B.
(0.37 MB DOC)

Figure S3 Variability of saccade amplitude gain as function of experimental and control sessions types. Grey background areas correspond to pre-injection sessions; white background areas to post-injection sessions.
(0.26 MB DOC)

Figure S4 Variability of relative saccade direction as a function of the experimental and control session type. Grey background areas correspond to pre-injection sessions; white background areas to post-injection sessions.
(0.26 MB DOC)

Figure S5 Log-log plot. (A) Log-log plot of average Lomb-Scargill power spectrum estimates, for response times sequences following GABA_A_ agonist (thick orange line) and GABA_A_ agonist (thin blue line) injections for Monkey A. Frequency f is cycles-per-block. (B) Same convention for monkey B.
(0.91 MB DOC)

Figure S6 Variability of response time. (A) Variability of response times as function of experimental session types for monkey C after injection in the right SEF. When the eye movement is produced ipsilaterally (full line box) or contralaterally (dotted line box) to the injection site. (B) same convention as A after injection in the left SEF. (C) same convention as A and B after injection in the Pre-SMA.
(0.39 MB DOC)
