# Supplementary material for: A Fragile Balance: Perturbation of GABA Mediated Circuit in Prefrontal Cortex Generates High Intraindividual Performance Variability
Source: PLoS One. 2009 Apr 21;4(4):e5208. doi: 10.1371/journal.pone.0005208 (PMC2668174; doi:10.1371/journal.pone.0005208)
Supplement: Text S1 — (0.02 MB DOC) [file pone.0005208.s001.doc]

**SUPPLEMENTARY MATERIALS:**

1. *Serial correlation*: A standard method of analyzing a sequence of data is to compute its serial correlation, otherwise known as the autocorrelation. The autocorrelation of a sequence at lag t is the correlation between all data in the sequence that are t time steps apart (e.g. Chatfield, 1996). The autocorrelation can be visualized by imagining the sequence of data duplicated, then shifted, and computing the correlation between all pairs of data where the two sequences overlap. There are strong and significant correlations between RTs and previous RTs up to 8 trials in the past. The correlations are highest for pre-injection blocks, and lowest for post-injection of GABA_A_ antagonist. The serial correlations between RTs and previous RTs were not affected by injection of GABA_A_ agonist.
2. *Spectral analysis*: Another method of analyzing time series is to estimate the power spectrum. Standard spectral analysis is most suited to series where all the data are regularly spaced in time, so again some adjustments are needed to use spectral analysis on the heterogeneous sequence of RTs. The Lomb–Scargle periodogram is a least-squares estimate of the power of sinusoidal components in the series. The Lomb–Scargle periodogram was used in the spectral analyses presented here using a Matlab implementation. The spectra were averaged across sessions and monkeys because the differences between the power spectra were fairly small. The averaged power of the data following GABA_A_ antagonist shows a Brownian-like spectrum (supplementary Figure 6 for monkey A and monkey B). Frequency f is expressed in cycles-per-block, so that for a block of 100 trials a frequency of 4, for example, has a “wavelength” of 100/4 = 25 trials.
